# Aurora-B knockdown inhibits osteosarcoma metastasis by inducing autophagy via the mTOR/ULK1 pathway

**DOI:** 10.1186/s12935-020-01674-1

**Published:** 2020-11-30

**Authors:** Xin Wu, Jia-ming Liu, Hong-hai Song, Qi-kun Yang, Hui Ying, Wei-lai Tong, Yang Zhou, Zhi-li Liu

**Affiliations:** 1 Department of Orthopedic Surgery, The First Affiliated Hospital of Nanchang University, No.17 Yong Wai Zheng Street, Donghu District, Nanchang, Jiangxi 330006 People’s Republic of China; 2 Spine & Spinal Cord Institute, Nanchang University, No.17 Yong Wai Zheng Street, Donghu District, Nanchang, Jiangxi 330006 People’s Republic of China

**Keywords:** Osteosarcoma, Aurora-B, The mTOR/ULK1 pathway, Autophagy, Metastasis

## Abstract

**Background:**

Autophagy plays an essential role in metastasis of malignancies. Although our studies showed that Aurora-B facilitate pulmonary metastasis in OS, the mechanism of Aurora-B kinase on autophagy and metastasis in OS has not been explored.

**Methods:**

Clinical-pathological parameters and follow-up information was collected in OS patients. Immunohistochemical staining was performed to detect Aurora-B and LC3 protein in OS tissues. Short hairpin RNA transfection was used to silence Aurora-B in OS cells. Real-time quantitative PCR (RT-qPCR) was performed to detect Aurora-B mRNA expression in OS cells. Aurora-B and autophagy related protein were measured by Western blot. Transmission electron microscopy and laser scanning confocal microscopy were performed to observe the formation of autophagosomes and autolysosomes. Migratory and invasive ability of OS cells were measured by Wound healing and transwell assays. Orthotopic xenograft model was used to evaluate the effect of autophagy mediated by Aurora-B inhibition on pulmonary metastasis of OS.

**Results:**

The elevated expression of Aurora-B protein in OS tissues negatively associated with the overall survival of OS patients. Further investigation has found that Aurora-B expression was negatively correlative with autophagy related protein LC3 in OS patient tissues. Knockdown Aurora-B stimulates autophagy and inhibits migratory and invasive ability of OS cells. Mechanistically, Aurora-B knockdown suppressed the mTOR/ULK1 signaling pathway and reactivation of the mTOR/ULK1 pathway decreased autophagy level. Furthermore, the inhibition effect of silencing Aurora-B on migration and invasion of OS was reversed by chloroquine and mTOR activator in vitro and vivo.

**Conclusions:**

Our results suggest that silencing of Aurora-B stimulate autophagy via decreasing mTOR/ULK1 and result in inhibiting OS metastasis. Targeted Aurora-B/mTOR/ULK1 pathway may be a promising treatment strategy for OS patients.

## Background

Osteosarcoma (OS), a highly aggressive tumor with a tendency to metastasize to the lung, is the most common malignant bone tumor in children and adolescents [1, 2], and metastasis to the lung is the leading cause of death in patients with OS [3]. Despite the combination of surgery resection with neoadjuvant chemotherapy strategies for OS, the 5 year overall survival rate has remained unchanged, at 65–70%, over the past few decades [4, 5]. In addition, the 5 year survival for metastatic disease is around 20%, highlighting the need for novel therapeutic targets. Therefore, elucidation of the molecular and cellular drivers involved in the pathogenesis of OS should facilitate the development of effective new strategies for the management of this malignancy.

Autophagy is an intracellular degradation process that removes and recycles damaged proteins and organelles to extend cell longevity. Numerous studies have shown that autophagy is exploited by tumor cells as a highly dynamic process to suppress initial stage of tumor in carcinogenesis by limiting chromosomal instability, restricting oxidative stress, and preventing intratumoral necrosis and local inflammation [6, 7]. Furthermore, in the early stages of cancer metastasis, autophagy may restrict neoplasm metastasis by suppressing tumor necrosis and inflammatory cell infiltration, and by reducing tumor-induced senescence. Studies have indicated that the induction of autophagy inhibits proliferation, invasion, and migration in bladder cancer and OS cells [8–10]. However, the underlying molecular regulatory mechanisms require further elucidation.

Aurora-B kinase, a member of the aurora kinase family, is a ubiquitously expressed serine/threonine kinase that phosphorylates histone H3 on Ser10 and variant centrosome protein A on Ser7 in early G2, resulting in the condensation of chromatin 18. Aurora-B participates in the regulation of the spindle assembly complex, chromosome segregation, and cytokinesis [11]. Therefore, silencing or loss of Aurora-B leads to defective chromosome segregation and polyploidy. Abundant evidence has shown that Aurora-B is expressed at high levels in various malignant tumors, and represents an important antitumor target [12–14]. Amplification or increased expression of Aurora-B has been shown to be associated with poor prognosis in various human malignant tumors [15–17]. Further, Aurora-B is a therapeutic target in non-small cell lung cancer refractory to anti-EGFR therapy [18].Our previous study indicated that the expression of Aurora-B is elevated in OS tissues and cell lines, and that silencing of Aurora-B inhibited the malignant phenotype of OS cells in vitro [19]. However, the mechanisms by which Aurora-B promotes OS pulmonary metastasis have not been fully elucidated to date.

In this study, we investigated the role, and potential underlying mechanisms, of Aurora-B in the pathogenesis of OS, and found that the expression of this kinase is negatively correlated with prognosis and autophagy in OS, which had not been shown before. Furthermore, Aurora-B silencing was shown to inhibit migration and invasion of OS cells by increasing the levels of autophagy mediated by mTOR inhibition. Our results identified a novel as a potential therapeutic target and prognostic biomarker in OS patients.

## Material and methods

### Tissues specimens and patients

Sixty-nine OS tissue samples were obtained by surgical biopsy from the First Affiliated Hospital of Nanchang University, China. Patients who had received radiotherapy or chemotherapy before undergoing biopsy were excluded. Donors included 29 females and 40 males, with a mean age of 26 years (range 5–72 years). All OS tissues were subject to pathological examination, and the expression of Aurora-B and LC3 was evaluated through IHC analysis. The clinical parameters are shown in Table 1; follow-up information was missing for 10 of these patients. Informed study protocols were completed in accordance with the declaration of Helsinki and “Guiding Opinions on the Treatment of Animals” in China. The medical ethics committee of the First Affiliated Hospital of Nanchang University has approved this experimental protocol (Jiangxi, China; NO. Y2019-126).

**Table 1:** Correlation of Aurora-B protein expression levels in OS tissues with clinical pathologic parameters

Variables	All cases	Aurora-B expression		*P* value
		Low	High	
Gender				
Male	40	19 (47.5%)	21 (52.5%)	0.328
Female	29	18 (62.1%)	11 (37.9%)	
Age				
≤ 20	33	19 (57.6%)	14 (42.4%)	0.631
> 20	36	18 (50%)	18 (50%)	
Location				
Femur/Tibia	53	27 (50.9%)	26 (49.1%)	0.569
Elsewhere	16	10 (62.5%)	6 (37.5%)	
Tumor size (cm)				
≤ 5	23	16 (69.6%)	7 (30.4%)	0.076
> 5	46	21 (45.7%)	25 (54.3%)	
Enneking staging				
I + IIA	40	26 (65%)	14 (35%)	0.031^*^
IIB + III	29	11 (37.9%)	18 (62.1%)	
LC3B expression				
Low	34	13 (38.2%)	21 (61.8%)	0.016^*^
High	35	24 (68.6%)	11 (31.4%)	

*P* value was calculated by Pearson’s Chi-Square test. *P < 0.05

### Histology and IHC

OS tissue samples were fixed in 4% paraformaldehyde for 20 min, embedded in paraffin, and sectioned to a thickness of ~ 3 µm. These slides were deparaffinized and rehydrated, and then treated with 0.2% Triton X-100PBS for 10 min and blocked with 3% hydrogen peroxide at room temperature for 20 min. These slides were autoclaved in 10 mM citric acid solution to enhance the antigen retrieval for 2 min. The samples were incubated with anti-Aurora-B (Abcam ab45145) and anti-LC3 (Cell signaling technology 2775) overnight at 4 °C. Samples were incubated with appropriate secondary antibodies for 30 min using Histostain Plus kits (Invitrogen, CA, USA). Pictures were captured with the microscope and determined by two pathologists blinded to the specimens.

### Cell culture

HOS and 143B cell lines were obtained from the Type Culture Collection of the Chinese Academy of Sciences, Shanghai, China. All these cells were cultured in DMEM medium (Gibco) supplemented with 10% fetal bovine serum (FBS, Gibco, 10099141C) with penicillin 100 U/mL and streptomycin 100 g/mL (Solarbio, Shanghai, China). All these cells were cultured at 37 °C with 5% CO2.

### Generation of AuroraB knockdown 143B and HOS cells

143B and HOS cells (1X104) were cultured in 35 mm cell-culture dish and were infected with 2X105 Lentivirus-Vector (MOI = 20) with Aurora-B (Lv-shAuroraB were inserted into lentivirus vector GV115 (GeneChem Co., Ltd., Shanghai, China), 5′-CCG GCTCCAAACTGCTCAGGCATAACTCGAGTTATGCCTGAGCAGTTTGGAGTTTTTG-3′) for 72 h and puromycin were incubated to the cells for screening. The efficacy of gene knockdown was determined by Western blot and qRT-PCR.

### Western blot analysis

Human osteosarcoma cells were lysed in RIPA buffer containing protease inhibitor (cocktail and PMSF) for 15 min on ice. Protein concentrations were measured by BCA Protein Assay kit (Thermo Fisher Scientific). Total cell lysates were electrophoresed by sodium dodecyl sulfate–polyacrylamide gel electrophoresis (SDS-PAGE) on 8%-15% gels and transferred onto PVDF membranes (Millipore). The membranes were blocked with 5% skim milk (BD) for 60 min at room temperature and incubated with primary antibodies overnight at 4 °C. Membranes were washed with 1X TBST 3 times and followed by incubation with secondary antibodies for 2 h. The immune complexes were visualized and measured with an ECL system (Bio-Rad, CA, USA) and the digital gel image analysis system (TANON). The primary antibodies included rabbit anti-human AuroraB (Abcam ab45145), anti-β-tubulin (Abcam ab179513), anti-SQSTM1/P62 (Cell signaling technology 5114), rabbit anti-LC3B (Cell signaling technology 2775), anti-p-mTOR(ser2448)(Cell signaling technology 2971), anti-mTOR(Cell signaling technology 2972), anti-AMPK (Cell Signaling Technology, 2532), anti-pAMPKα (Thr172) (Cell Signaling Technology, 2535), anti-ULK1 (Cell Signaling Technology, 8054) and anti-Pulk1 (Ser555) (Cell Signaling Technology, 5869), mouse anti-human GAPDH (Origene TA802519) and anti-MMP2 (Origene TA806846S). The secondary antibodies included HRP-conjugated Affinipure Goat Anti-Mouse IgG (H + L) (proteintech SA00001-1) and HRP-conjugated Affinipure Goat Anti-Rabbit IgG (H + L) (proteintech SA00001-2).

### Quantitative real-time PCR analysis

The total RNA was extracted from the osteosarcoma cell samples by Trizol (Solarbio, Shanghai, China), with the Revert Aid First Strand cDNA Synthesis Kit (Thermo fisher, USA) reverse-transcribed into cDNA. Q-PCR reactions were performed by the TaqMan™ Fast Advanced Master Mix (Thermo fisher, USA), GAPDH was used as a control. The Aurora-B sequence is Forward (5′ → 3′) AGAAGGAGAACTCCTACCCCT, Reverse (5′ → 3′) CGCGTTAAGATGTCGGGTG, GAPHD sequence is (Forward (5′ → 3′)) CCACCCATGGCAAATTCCATGGCA, Reverse (5′ → 3′) TCTAGACGGCAGGTCAGGTCCACC.

### LC3 fusion assay

In order to track autophagosomes, HOS and 143B cells were transfected with lentiviral vectors harboring GFP-RFP-LC3 (GeneChem Co., Ltd., Shanghai, China) to obtain cells with stable expression of the GFP-RFP-LC3B protein. Then, these cells were treated with Lv-shAurora-B and control groups for the indicated duration. Images were captured by laser scanning confocal microscopy (ZEISS/LSM 800, Germany) to observe the fluorescence spots mark autophagosomes in these cells.

### Transmission electron microscopy (TEM)

In brief, 143B and HOS cells were collected, fixed with 2.5% glutaraldehyde and encased. Ultrathin 60–80 nm sections were prepared with an Ultramicrotome (Leica UC7; Germany) and stained with uranyl acetate (15 min) and Reynolds lead citrate (15 min). The images were captured by a transmission electron microscope (HITACHI HT7700; Japan).

### Transwell migration and invasion assay

The Millipore 8 µm 24-well transwell chamber (Millipore) was used in the osteosarcoma cells migration and invasion. Cells (3 × 10^4^ cells per well) were loaded on FBS-free DMEM in the upper chamber of well coated with or without Matrigel (100 µl; 1:20 dilution; BD Biosciences), The lower chambers were filled with 500 μl complete medium mixed with 12 µm CQ or 2 µm MHY-1485 and a corresponding dose of PBS as control. After 12 h, the lower chambers mixtures were removed and replaced with 500 μl new complete medium. Another 12 h later, cells on the upper surface of the well were removed, and invasion cells passed through the well on the bottom were stained with 1% crystal violet. Cells in six randomly selected fields were counted and photographed (magnification 10X).

### Wound healing assays

143B and HOS cells were grown to confluence in 6-well plates in the density of 5–8 × 10^6^ cells per well. When the cells were grown to 90% confluence, we used 20 µl pipette tip to scratch wounds through the center of the plate. The cells were washed three times with PBS to remove the floated cells and then incubated with 1% FBS MDEM mixed with 12 µm CQ or 2 µm MHY-1485 and a corresponding dose of PBS as control at 37 °C for 8 h. The mixtures were removed and replaced by new 1% FBS MDEM. Images were captured at different time points (0 and 24 h), and the migration distance was measured by ImageJ compared with the time zero.

### In vivo assay

All experimental protocols were approved by the Institutional Animal Care and Use Committee of Nanchang University (Jiangxi, China; NO. Y2019-126). We purchased female BALB/C nude mice at 6 weeks of age from the Nanjing Biomedical Research Institute of Nanjing University (NBRI, Nanjing, China); mice were housed in the SPF (Specific Pathogen Free) Transgenic Animal Facility of Nanchang University. The protocol for generation of a spontaneous metastasis/orthotopic osteosarcoma mouse model was applied as reported in our previous study [20]. The mice were randomly divided into four groups (Ctrl group, AZD2811 groups, ADZ2811 + 3BDO group, and AZD2811 + CQ groups; n = 6 in each group). Drugs (normal saline, AZD2811 (150 mg/kg), ADZ2811 (150 mg/kg), and 3BDO (80 mg/kg) mixture, AZD2811 (150 mg/kg), and CQ (80 mg/kg) mixture) were separately administered to mice via intraperitoneal injection, twice a week. After 6 weeks, the tumors were dissected and fixed in 10% formalin. The lung tissues were dissected to evaluate pulmonary metastasis by optical microscope using the Vivo imaging system (Berthold LB983; Germany). The tissues were fixed in 10% formalin for further detection.

### Statistical analysis

All these quantitative data were presented as the mean ± SD. Student's t-test was performed for two-sample analysis, and one-way ANOVA was performed for multiple-sample analysis by using GraphPad Prism 7 software. *P* < 0.05 was considered a statistically significant difference.

## Results

### Aurora-B expression was negatively correlated with prognosis in OS.

**Fig. 1:**
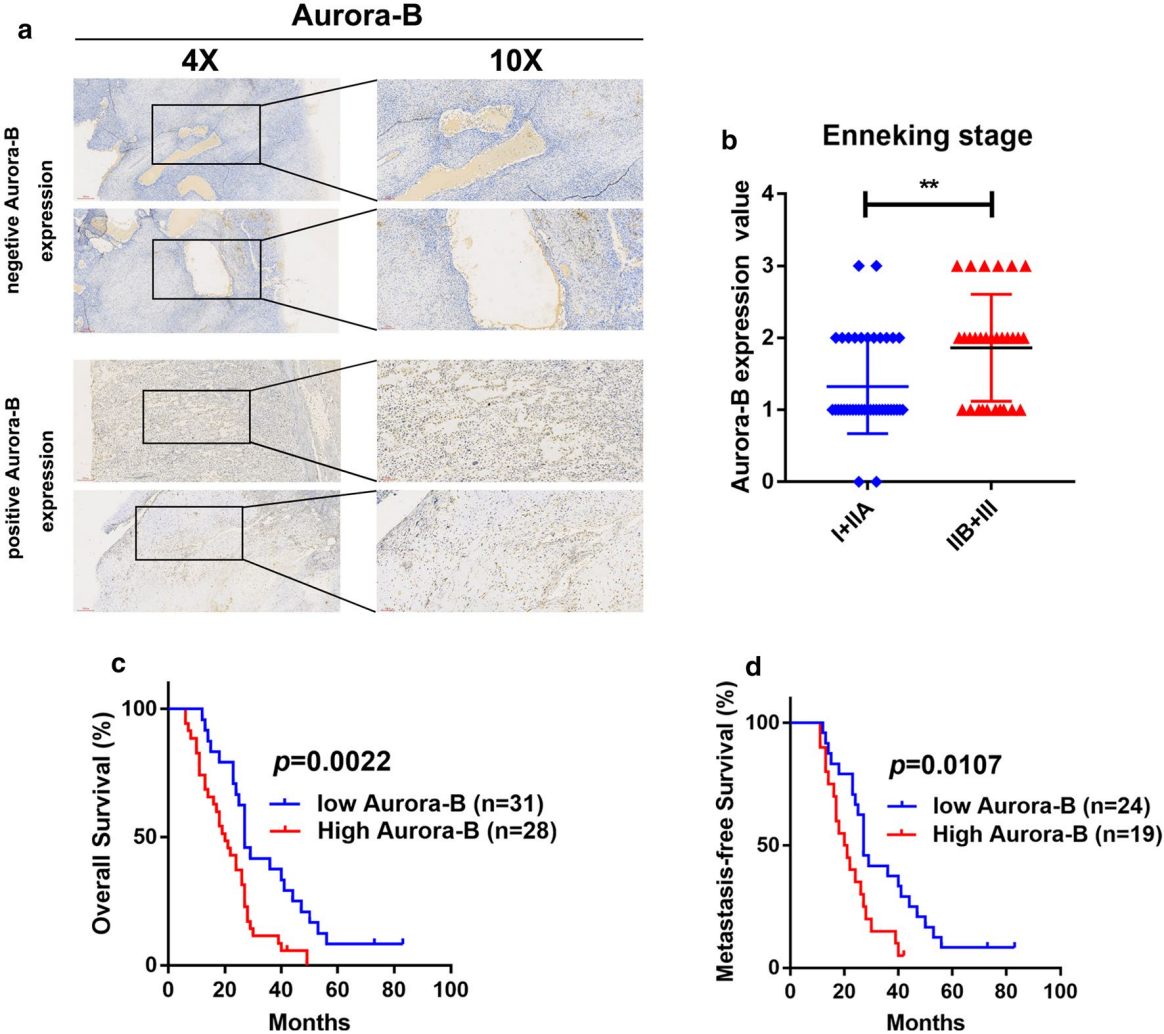
Aurora-B expression was negatively correlated with prognosis in OS. Tissue samples and data for clinical-pathological parameters were collected from 69 OS patients. The expression of Aurora-B protein in tumor tissues were detected by immunohistochemical staining (**a**). Statistical analysis of Enneking stage classification (**b**), **P < 0.01 in 69 OS patients, among them, there are 40 cases of I + IIA and 29 cases of IIB + III. **c** *P *< 0.01, **d** *P* < 0.05 Kaplan–Meier survival analysis according to Aurora-B expression in 59 patients to be followed up

To investigation the relationship of Aurora-B expression with prognosis in OS. We examined the expression of Aurora-B by immunohistochemical staining and collected the follow-up information from OS patients. We found that the expression of Aurora-B protein is closely correlated with Enneking stage (Table 1; Fig. 1a, b) (*P* < 0.01). Moreover, Kaplan–Meier analysis showed that high levels of Aurora-B expression were negative correlated with poor overall survival (Fig. 1c) (*P* < 0.01) and lower metastasis-free survival (Fig. 1d) (*P* < 0.01) in these OS patients. These results collectively indicate that the expression of Aurora-B protein was negatively correlated with prognosis in OS.

### Aurora-B knockdown induces autophagy in OS

**Fig. 2:**
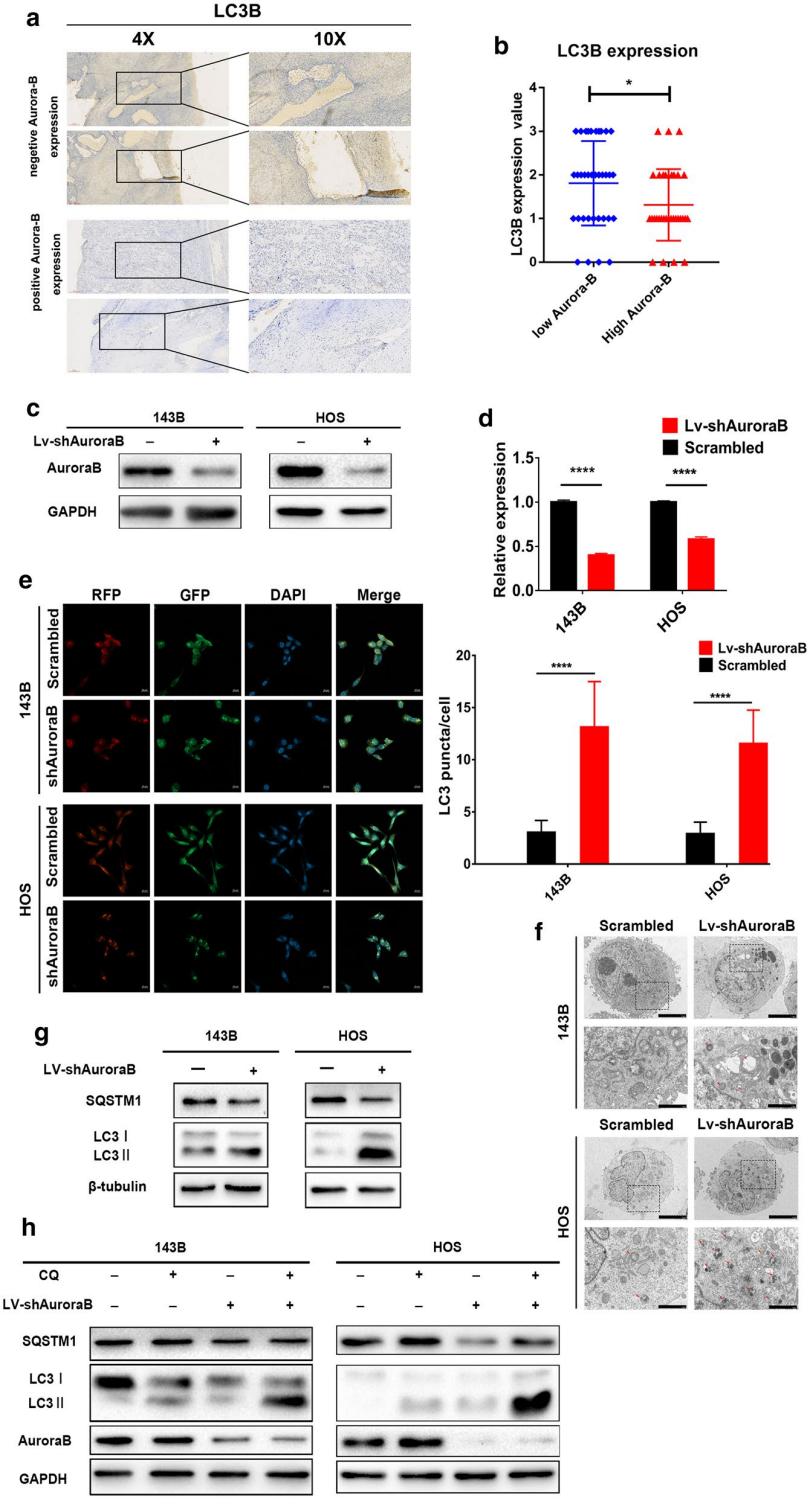
Aurora-B knockdown induces autophagy in OS. The expression of LC3 protein in tumor tissues were detected by immunohistochemical staining (**a**), and analyzed by staining scores was performed in 69 OS patients. There are 37 cases of low Aurora-B expression and 32 cases of high Aurora-B expression. **b** *P < 0.05.143B and HOS cells were infected with shAurora-B and scrambled lentivirus. Aurora-B expression in 143B and HOS cells was knocked down using shAurora-B lentivirus, and analyzed by western blotting (**c**) and qRT-PCR (**d**). ****P < 0.01. The RFP-GFP-LC3 fusion assay was performed and cells were visualized by laser scanning confocal microscopy (**e**); fusion numbers were quantified by randomly selecting three views per slide. ****P < 0.01, Scale bars: 20 μm. Autophagosomes and autolysosomes were observed with a transmission electron microscope (**f**). Scale bars: 5 μm (left pictures) and 1 μm (right pictures). The protein level of LC3-I, LC3-II, and SQSTM1 were analyzed by western blotting (**g**). Infected cells were treated with 10 μM CQ (chloroquine diphosphate) for 24 h. The total level of LC3-I, LC3-II, and SQSTM1 proteins were used to detect the autophagic flux (**h**)

To investigate the correlation between Aurora-B and autophagy, we first applied immunohistochemical staining to examine the Aurora-B and LC3 expression in OS tissues. In the tissues with Aurora-B-positive expression, LC3 protein was poorly expressed, and the opposite was observed in the tissue samples that tested negative for Aurora-B expression (Table 1; Fig. 2a, b) (*P* < 0.05). These results suggested that a potential correlation between Aurora-B and autophagy in OS. Further investigate the effect of Aurora-B on autophagy in OS cells, we knocked down endogenous Aurora-B with shRNA (short-hairpin RNA) in 143B and HOS cells by lentiviral infection (Fig. 2c, d). The RFP-GFP-LC3 fusion assay was performed to observe the formation of autophagosomes and autolysosomes in OS cells. The number of yellow LC3 puncta (representing autophagosomes) and red LC3 puncta (representing autolysosomes) were both higher in Aurora-B silenced cells than in cells infected with scrambled lentivirus, which represented the negative control (Fig. 2e). Similar results were observed by transmission electron microscopy (Fig. 2f). Furthermore, the ratio of LC3-II to LC3-I was significantly increased, whereas p62 expression was decreased, in cells with Aurora-B-knockdown relative to that in cells infected with scrambled lentiviruses (Fig. 2g). The results indicate that inhibition of Aurora-B enhanced autophagy in OS cells. Furthermore, to distinguish whether the accumulation of LC3 is due to the induction of autophagy or the depletion of autophagy flux, cells were treated with the specific autophagy inhibitor chloroquine (CQ), which inhibits autophagosome-lysosome fusion, thus preventing LC3 degradation. As shown in Fig. 1f, the LC3 II protein level was significantly increased following treatment with CQ in both control and Aurora-B-silenced cells compared with that in cells that were not treated with CQ (Fig. 2h). These results indicate that Aurora-B knockdown more likely enhances autophagic flux than inhibits autophagy in OS cells.

### Silencing Aurora-B induces OS cells autophagy by suppressing the mTOR/ULK1 pathway

**Fig. 3:**
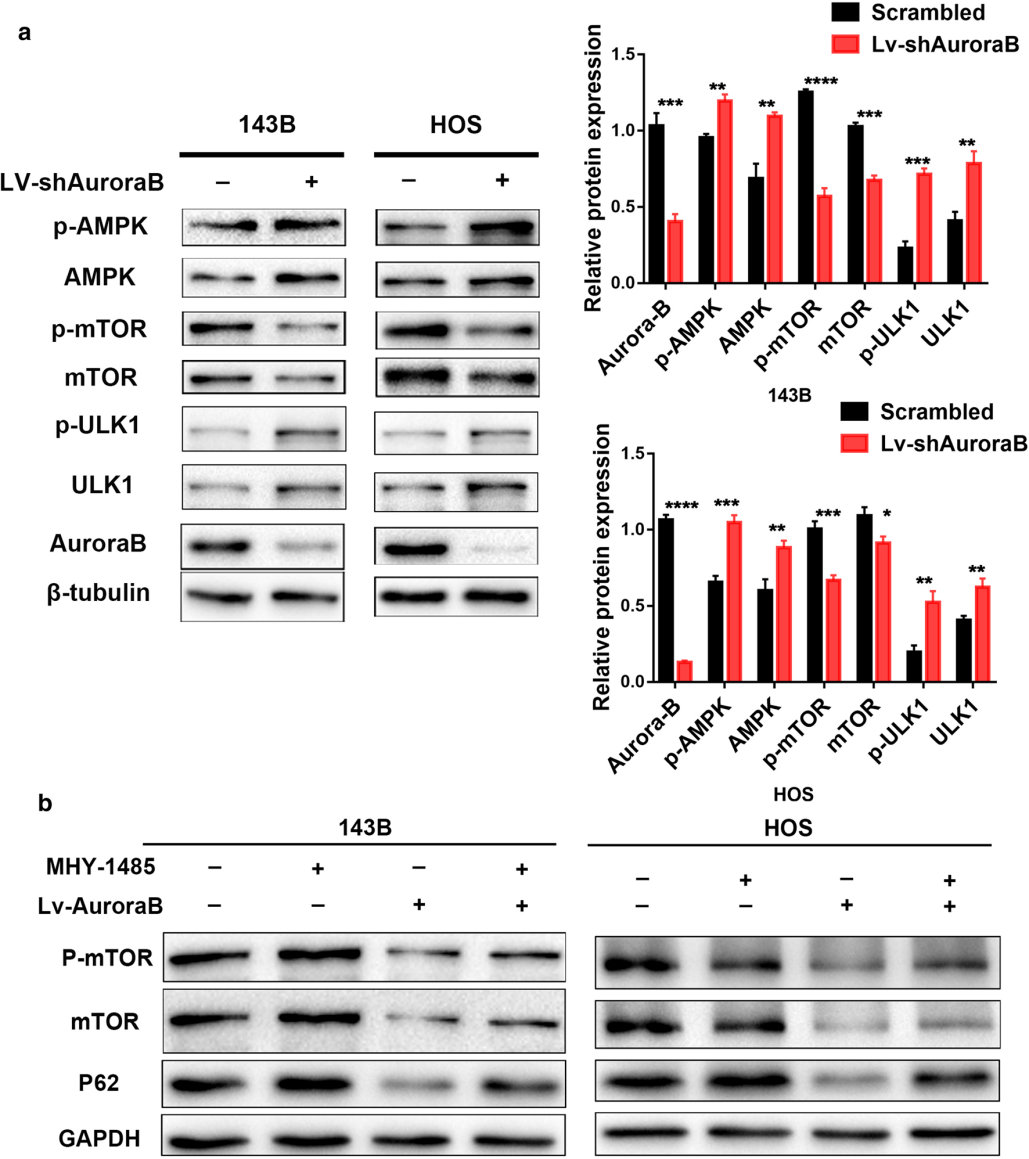
Silencing Aurora-B induces OS cells autophagy by suppressing the mTOR/ULK1 pathway. Aurora-B silencing and corresponding contrast OS cells were used to measure examine the mTOR/ULK1 pathway signaling and autophagy. The protein levels of AMKP, p-AMPK, mTOR, p-mTOR, ULK1, and p-ULK1 in Aurora-B B-knocked down 143B, and HOS cells were analyzed by western blotting (**a**). Treated Following treatment of these OS cells with or without MHY-1485 (2 μM, 3 h) in these OS cells, or not, the expression of P62p62, p-mTOR, and mTOR was detected investigated by western blotting (**b**)

The mTOR/ULK1 pathway is known to regulate autophagy, and the inactivation of this pathway has been shown to enhance autophagy levels [21, 22]. AMPK is a critical sensor of stress and energy metabolism that negatively regulates mTOR, activating autophagy initiation factor ULK1 and promoting autophagic flux [23, 24]. To explore the molecular mechanisms involved in the enhancement of autophagy by Aurora-B knockdown, we examined the expression of proteins involved in the mTOR/ULK1 pathway in Aurora-B-knockdown 143B and HOS cells. Interestingly, as shown in Fig. 3a, the phosphorylation levels of AMPK and ULK1 were upregulated and those of mTOR were downregulated in Aurora-B knockdown 143B and HOS cells, which was consistent with the observed changes in total AMPK, ULK1, and mTOR levels. These results suggest that Aurora-B knockdown enhances autophagy by decreasing mTOR/ULK1 pathway activity. Recent evidence has additionally demonstrated that the suppression of the malignant phenotype of OS cells by Aurora-B silencing might be achieved via autophagy-mediated activation of the mTOR/ULK1 signaling pathway. Thus, to further investigate the role of the mTOR/ULK1 pathway during Aurora-B silencing-induced autophagy, we investigated the expression of the autophagy-related protein p62 after treatment with MHY-1485, a well-known mTOR activator [25], or not, in Aurora-B-knockdown or scrambled OS cells. In the Aurora-B-knockdown OS cells, MHY-1485 treatment activated mTOR/ULK1 signaling and increased p62 levels. In the scrambled groups, after treatment with MHY-1485, the expression of p62 and phosphorylation of mTOR increased (Fig. 3b). These results reveal that inhibition of Aurora-B induced autophagy in OS cells via decreasing the mTOR/ULK1 pathway.

### Aurora-B knockdown-induced autophagy inhibits migration and invasion in OS cells

**Fig. 4:**
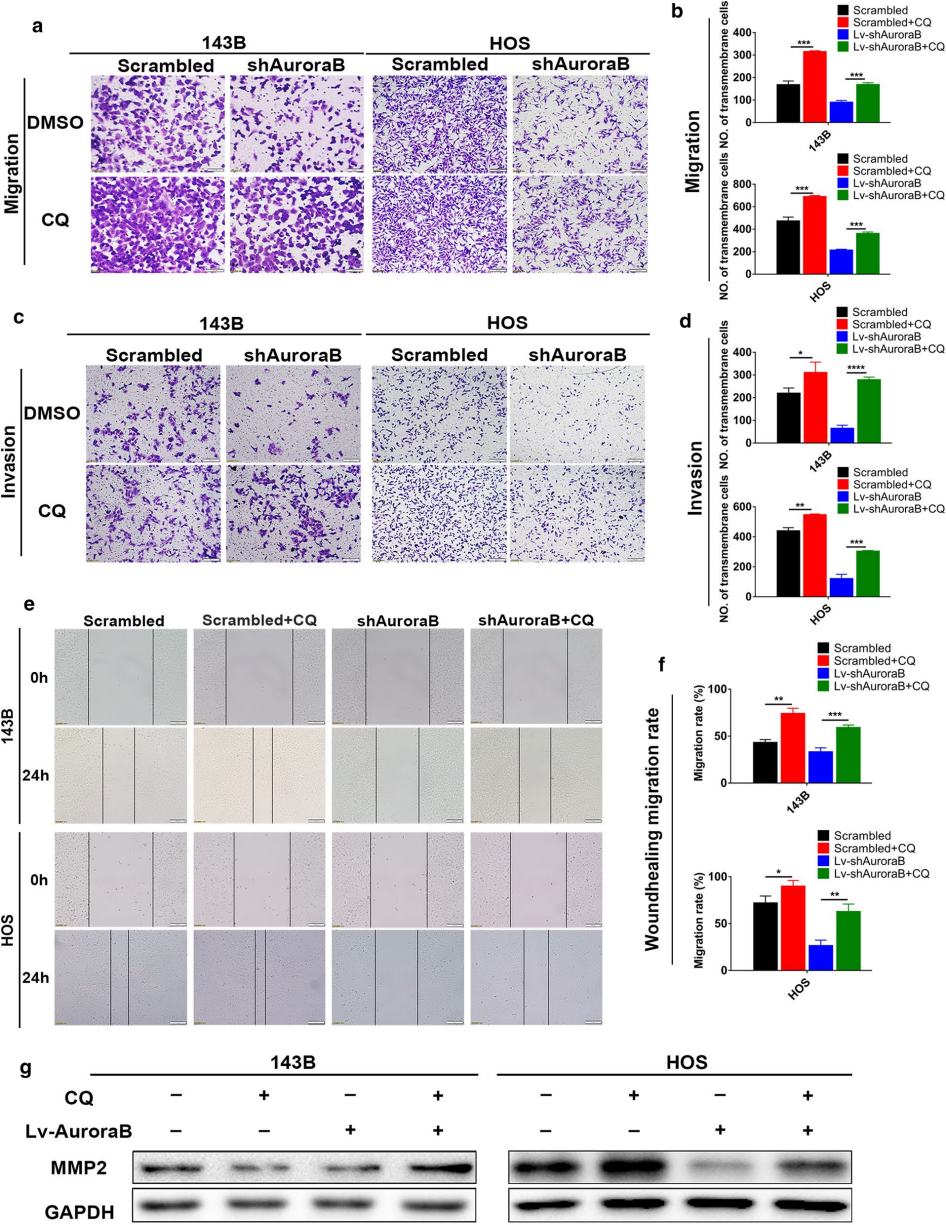
Aurora-B knockdown-induced autophagy inhibits migration and invasion in OS cells. The migration and invasion ability of Aurora-B-knockdown 143B and HOS cells treated with or without CQ (12 μM, 12 h) were examined by transwell migration (**a**, **b** ***P < 0.01, Scale bars: 100 μm) and invasion (**c**, **d** *P < 0.05, ****P < 0.01, ***P < 0.01,**P < 0.01, Scale bars: 100 μm) assays in vitro. Wound healing assay was performed with scrambled and shAurora-B 143B and HOS cells treated with CQ (12 μM, 12 h) for 24 h or not (**e**, **f** **P < 0.01, ***P < 0.01, *P < 0.05, Scale bars: 100 μm). MMP2 protein expression of Aurora-B-knockdown cells treated with CQ (10 μM, 24 h) or not was analyzed by western blotting (**g**)

Autophagy is known to be involved in tumor progression [26, 27]. In order to elucidate the effect of Aurora-B inhibition induced autophagy on the metastasis of OS cells, wound healing, transwell migration and invasion assays were performed to detect migration and invasion ability of OS cells. We surprisingly found that Aurora-B inhibition significantly decreased the motility and invasive ability of 143B and HOS cells. After treatment with CQ, the attenuated ability could be reversed. (Fig. 4a–f). These findings indicate that autophagy plays an essential role in the process of Aurora-B affecting the ability of motility and invasive in OS cells. Autophagy is reported to inhibit cell migration by accelerating the degradation of MMP family proteins [26, 27], therefore, we sought to determine the MMP2 protein levels in Aurora-B-knockdown OS cells with or without CQ treatment. Results showed that the suppression of MMP2 expression due to Aurora-B knockdown could be restored by CQ treatment (Fig. 4g). These findings demonstrate that inhibitio of Aurora B suppresses the migration and invasion of OS cells by stimulating autophagy.

### Activation of the mTOR/ULK1 pathway reverses the effect of Aurora-B inhibition on migration and invasion of OS cells

**Fig. 5:**
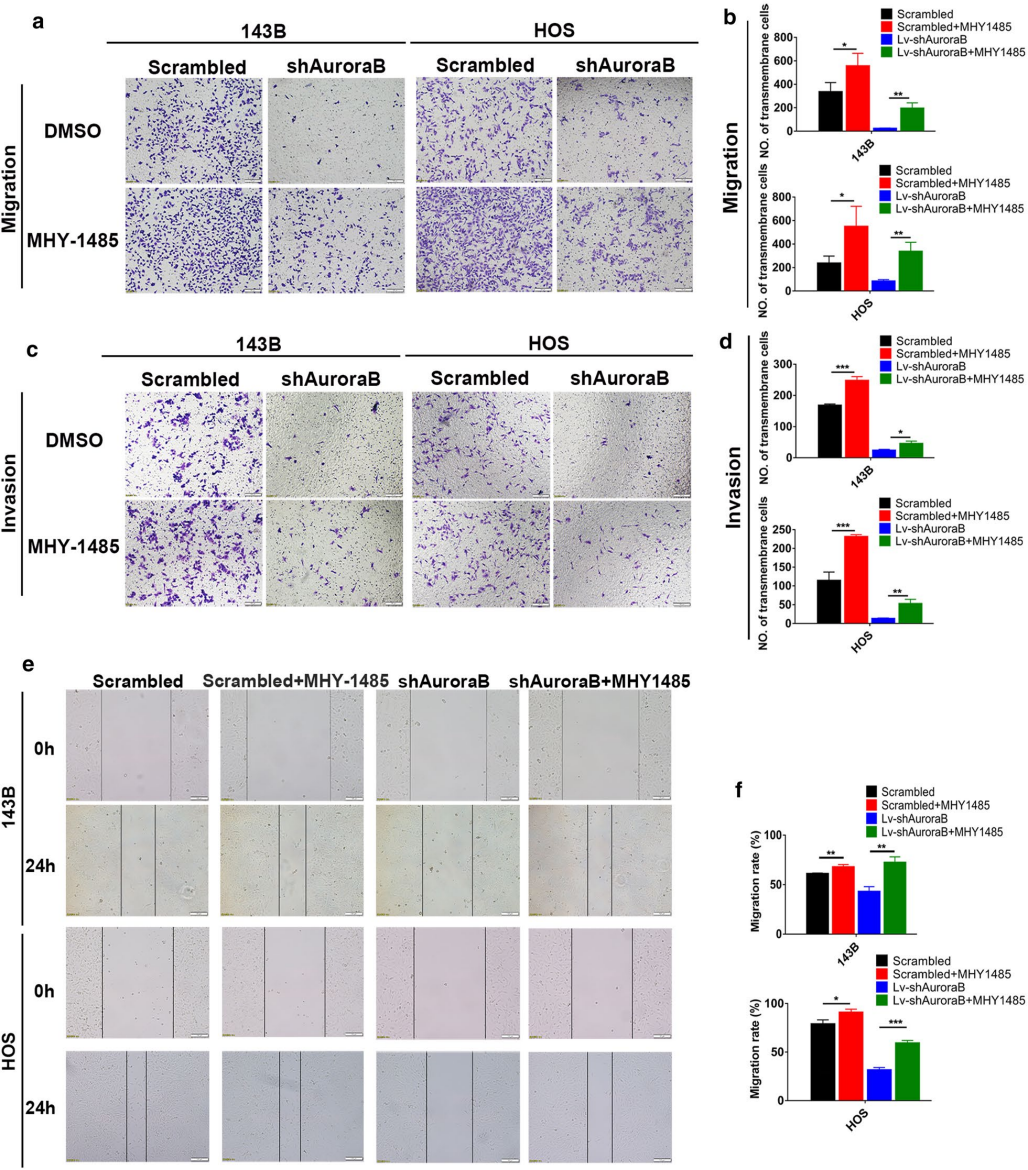
Activation of the mTOR/ULK1 pathway reverses the effect of Aurora-B inhibition on migration and invasion of OS cells. shAurora-B- and scrambled lentivirus-transduced 143B and HOS cells were used to detect migration and invasion. Transwell migration (**a**, **b** *P < 0.05, **P < 0.01, Scale bars: 100 μm) and invasion (**c**, **d** ***P < 0.01,*P < 0.05, **P < 0.01, Scale bars: 100 μm) assays were conducted for these cells following treatment with MHY-1485 (2 μM, 12 h) or not for 24 h. Wound healing assay (**e**, **f** **P < 0.01, *P < 0.05, ***P < 0.01, Scale bars: 100 μm) was performed in cells treated with MHY-1485 (2 μM, 12 h) for 24 h or not

To confirm whether the mTOR/ULK1 pathway contributes to Aurora-B-induced suppression of the malignant phenotype in OS cells, we performed wound healing, transwell migration, and invasion assays to evaluate the migration and invasion ability of Aurora-B-knockdown OS cells treated with MHY1485 or not. The results revealed that the inhibition of migration and invasion ability induced by Aurora-B knockdown could be reversed by MHY1485 treatment (Fig. 5a–f). And it indicates that activation of the mTOR/ULK1 pathway reverses the induction of Aurora-B knockdown-induced autophagy, which inhibits migration and invasion in OS cells.

### Aurora-B knockdown inhibits metastasis via mTOR-mediated autophagy in vivo

**Fig. 6:**
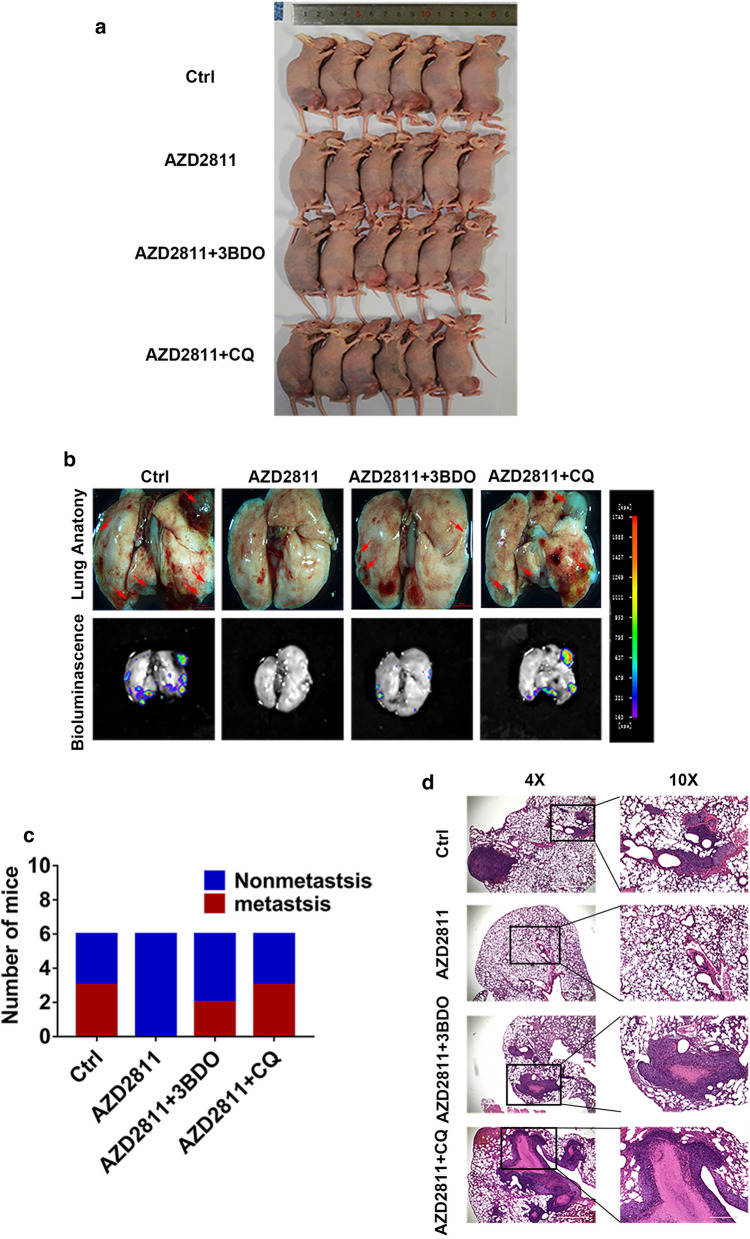
Aurora-B knockdown inhibits metastasis via mTOR-mediated autophagy in vivo. 143B cells stably expressing firefly luciferase were inoculated into the tibia of female BALB/C nude mice (n = 6) to establish an orthotopic model of spontaneous OS (**a**). After 6 weeks, mice were dissected and luminescence signals were detected (**b**). Then, the number of mice with metastases was counted (**c**). Representative H&E staining sections of the lungs were visualized by microscopy (**d**). Scale bars: 1000 μm (left images) and 400 μm (right images)

In order to investigate how the inhibition of Aurora-B affects the metastasis of OS, we established an orthotopic xenograft model using the luciferase-labeled 143B cell line (Fig. 6a). Bioluminescent imaging analysis revealed that the number of metastatic foci in the lung was significantly decreased when treated with AZD2811, a specific Aurora-B inhibitor in these mice models. Furthermore, 3BDO, a new mTOR activator, or CQ injection reversed these effects. H&E staining and lung anatomy confirmed this result (Fig. 6b–d). These results suggested that Aurora-B knockdown inhibits OS metastasis via mTOR-mediated autophagy in vivo.

## Discussion

Aurora-B, a serine/threonine kinase, plays a vital role in a variety of biological behaviors in cells, such as mitosis and the cell cycle [28]. Recent evidence has demonstrated that Aurora-B accelerates the progression of lung cancer [12], gastric cancer [29], prostate cancer [30], and OS [27]. Previously, we found that Aurora-B positive expression rate is increased in human OS tissues with pulmonary metastasis compared with that in non- metastasis tissues [31]. Here, to further examine the role of this kinase in OS metastasis and autophagy, we investigated the prognostic value of Aurora-B expression in 59 OS patients, and evaluated this kinase relationship with LC3 protein expression. We demonstrated that patients with high Aurora-B expression are likely to have a poor prognosis and low LC3 expression. In our previous study, Aurora-B was shown to partly activate migration and invasion via regulation of the NF-κB pathway and induction of MMP2 expression in OS [32]; however, the mechanism underlying Aurora-B-induced metastasis in OS requires elucidation. Interestingly, in the present study, we initially found that the effect of Aurora-B on OS invasion and metastasis could be regulated by mediating autophagy via the mTOR/ULK1 pathway; these findings had not been reported before and extended those of our previous study and confirmed the notion that Aurora-B knockdown suppresses autophagy-mediated OS metastasis via the mTOR/ULK1 pathway, and represents a useful biomarker of OS prognosis.

Autophagy, a complex biological behavior, plays a dual role in tumors such as OS [33]. In the early stages of tumorigenesis, autophagy can function as a tumor suppressor by inhibiting chromosomal instability, limiting oxidative stress, and inducing autophagic cell death to hinder metastasis and enhance the efficacy of chemotherapeutic drugs. In the later stages of tumorigenesis, autophagy can also function as a tumor activator by promoting metabolism and anoikis resistance, and maintaining homeostasis in tumor cells to promote metastasis and cancer progression [34]. In the present study, we investigated the potential role of Aurora-B in autophagy regulation, and found that a potential correlation between Aurora-B and autophagy in OS tissues and inhibition of Aurora-B could enhance autophagy in OS cells. Further investigation revealed that the use of chloroquine to inhibit autophagy activation promotes cell migration and invasion. This is consistent with the effect of autophagy on metastasis, as observed by Zhang et al. [35], and Liu et al. [36]. In contrast, Liu et al. found that microRNA-210-5p promotes metastasis by suppressing PIK3R5-induced autophagy via the mTOR pathway [37]. The differences in our conclusions may result from the method of autophagy inhibition: chloroquine suppresses autophagy by blocking autophagosome-lysosome fusion. Nevertheless, it is not an entirely specific autophagy inhibitor [38]. Specific autophagy inhibitors seem to be more suitable for the inhibition of autophagy, e.g. via RNA silencing or gene-specific targeting technologies to knockdown genes encoding autophagy-relevant components (e.g., ATG5, ATG7, or BECN1).

Autophagy is regulated by several signaling pathways, including mTOR, AMPK, and PKA [39–41]. Cao et al. found that the activation of autophagy is regulated by the AMPK/mTOR/ULK1 pathway in triple-negative breast cancer cells [24], whilst Zhang et al. demonstrated that thymoquinone inhibits metastasis in renal cell cancer cells by inducing autophagy via the AMPK/mTOR/ULK1 signaling pathway [42]. Emerging evidence demonstrates that the AMPK/mTOR/ULK1 signaling pathway is a critical regulator of tumor autophagy, and participates in the progressions of numerous tumor types. In this study, we found that the phosphorylation and total levels of AMPK and ULK1 expression were upregulated and those of mTOR were downregulated in Aurora-B-knockdown cell lines 143B and HOS. Further, because mTOR is well-known to regulate autophagy, we speculated that Aurora-B knockdown enhances autophagy by inhibiting the mTOR/ULK1 signaling pathway. In our previous study, we demonstrated that Aurora-B alters the malignant phenotype of OS cells partly via the PI3K/Akt/NF-κβ pathway [43], however, the underlying mechanism remains to be understood. Herein, we found that Aurora-B silencing inhibits migration and invasion of OS cells, whereas reactivation of mTOR phosphorylation suppresses Aurora-B knockdown-induced autophagy and reverses the inhibition of migration and invasion in OS cells. A similar phenomenon was observed in vivo. Our study illustrates the role of the mTOR/ULK1 signaling pathway in Aurora-B-knockdown-induced phenotype in OS.

## Conclusion

**Fig. 7:**
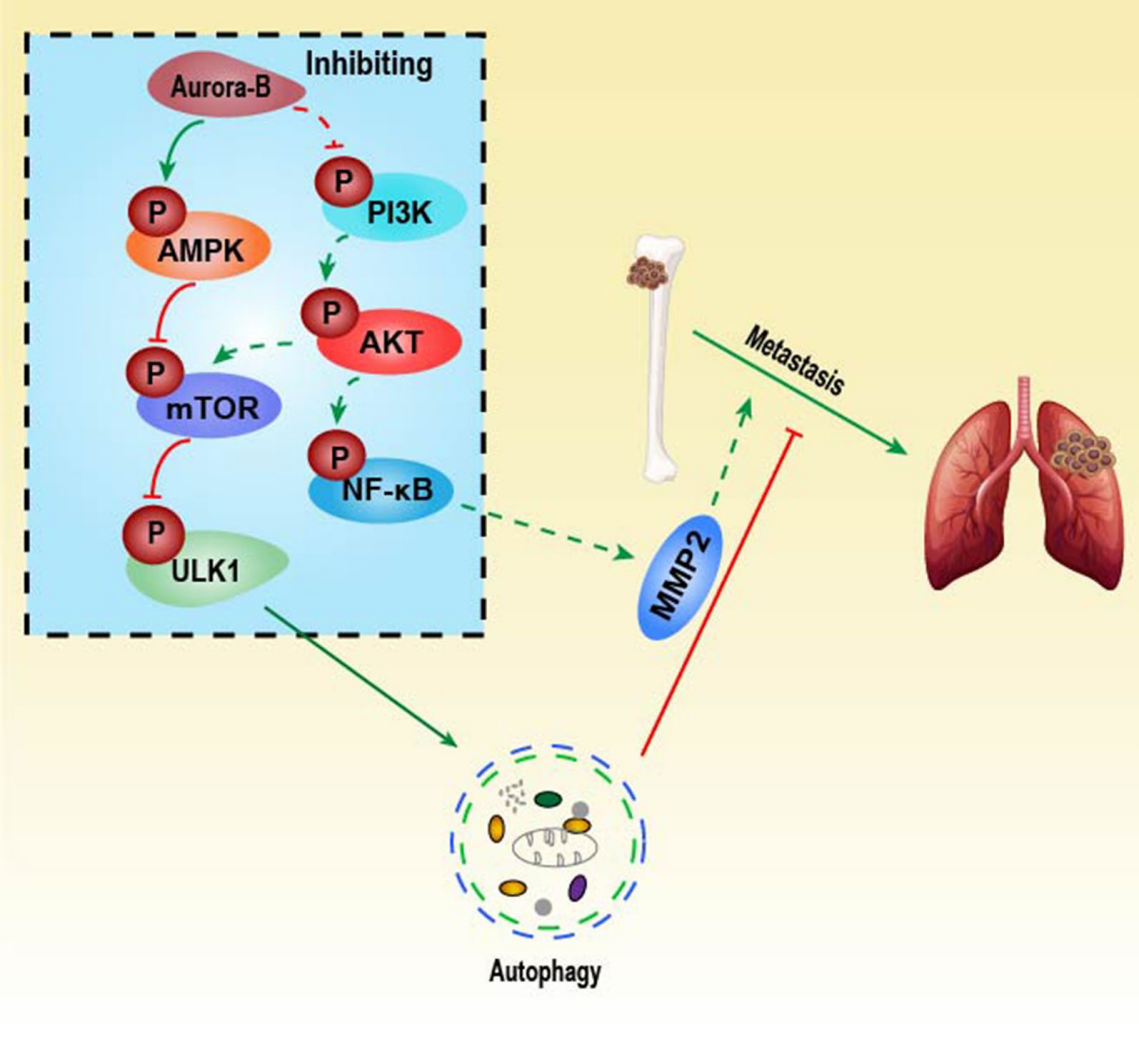
Schematic model of the mechanism by which Aurora-B silencing induces autophagy to suppress metastasis in OS

As shown in the Fig. 7, the findings indicated that silencing of Aurora-B stimulate autophagy via decreasing mTOR/ULK1 signaling pathway and result in inhibiting OS metastasis, this Aurora-B/mTOR/ULK1 axis may serve as a potential therapeutic target and prognostic marker for OS.

## Data Availability

Please contact corresponding author for data requests.

## Abbreviations

Aurora-B: Aurora kinase B
OS: Osteosarcoma
shRNA: Short hairpin RNA
IHC: Immunohistochemistry
qPCR: Quantitative PCR
FBS: Fetal bovine serum
MOI: Multiplicity of Infection
HCA: Hierarchical cluster analysis
TEM: Transmission electron microscopy
CQ: Chloroquine diphosphate
EMT: Epithelial-mesenchymal transition
